# Elusive effects of legalized wolf hunting on human-wolf interactions

**DOI:** 10.1126/sciadv.adu8945

**Published:** 2025-08-20

**Authors:** Leandra M. Merz, Bernhard Clemm von Hohenberg, Nicolas T. Bergmann, Jeremy T. Bruskotter, Neil H. Carter

**Affiliations:** ^1^ Department of Geography, San Diego State University, San Diego, CA, USA.; ^2^ School for Environment and Sustainability, University of Michigan, Ann Arbor, MI, USA.; ^3^ GESIS Leibniz Institute for the Social Sciences, Cologne, Germany.; ^4^ College of Agricultural, Human, and Natural Resource Science, Washington State University, Pullman, WA, USA.; ^5^ Department of Plant Sciences, University of Idaho, Moscow, ID, USA.; ^6^ School of Environment and Natural Resources, The Ohio State University, Columbus, OH, USA.

## Abstract

Expanding gray wolf (*Canis lupus*) populations in Europe and North America contribute to increased risks of livestock predation, which can threaten human livelihoods and lead government agencies to target wolves for lethal removal. Public wolf hunting is a highly contentious strategy for mitigating these risks, yet few empirical studies examine its effectiveness in doing so. Using difference-in-differences and structural equation modeling of data from the northwestern US between 2005 and 2021, we analyzed impacts of wolf hunting on livestock predation by wolves and government removal of wolves in the same year and with a 1-year time lag while controlling for social and environmental variables. We found that public wolf hunting had a small negative effect on livestock predation but had no effect on government lethal removal of wolves in the same or subsequent years. Our findings challenge the assumption that wolf hunting is an effective management strategy for reducing livestock predation and lethal removal.

## Teaser

Small or no effects suggest that wolf hunting is not an efficient approach to reducing negative human-wolf interactions.

## INTRODUCTION

Large carnivores occupy areas with livestock all over the world. By killing or harassing livestock on these shared landscapes, carnivores pose a heterogeneous but sometimes substantial risk to human interests and livelihoods (*1*). Managing the risks is a major, and growing, challenge (*2*). Human societies increasingly have diverse, sometimes conflicting, expectations and priorities regarding wildlife (*3*, *4*). These disparate viewpoints shape policy toward carnivores and may lead to gridlock or rapid policy shifts (e.g., “predator pendulum” where management rapidly shifts between strategies that heavily persecute wolves and others that heavily protect wolves)—none of which is conducive to sustainable management or conservation outcomes (*5*–*7*). Yet, ongoing range expansions of certain carnivore species are likely to exacerbate the conflict among humans regarding how carnivores should be managed.

A subject of intense, and timely, scrutiny is the management of gray wolf (*Canis lupus*) populations in North America and Europe. Gray wolves have rebounded since policies to conserve them were enacted during the 1960s and 1970s (*8*, *9*). They appear to thrive as long as prey is available and human-induced mortality is limited (*9*, *10*). As wolves reoccupy their former range, negative interactions between people and wolves have increased. These interactions have prompted calls to manage wolf populations using lethal actions (*11*). Notably, some government agencies facilitate public hunting of wolves as a means of reducing livestock losses from wolf predation [e.g., (*12*)]. If effective at reducing livestock depredation, then hunting wolves may also reduce the need for expensive lethal removal of chronically depredating wolves by government agencies (*13*). Although wolf hunts are increasingly used to manage wolves, they can be highly controversial, characterized by divisive discourse on the ethics, ecological effects, and societal impacts of hunting wolves (*14*, *15*). Therefore, empirical evidence that legalizing wolf hunting reduces negative impacts of wolves is needed to justify its use as a risk-reduction tool (*16*–*18*).

Despite the polarization over wolf hunting, few studies have quantified whether it demonstrably reduces livestock depredations and associated lethal removals by government agencies (*19*). Those studies often suggest that legalizing wolf hunting does not reliably and meaningfully reduce livestock predation unless all or most wolves in a given area are removed (*19*, *20*)—an outcome that contravenes policies in place to protect wolves. Kutal *et al.* (*21*) examined wolf-livestock interactions in Slovakia and found no evidence that livestock predation decreased following wolf hunting. Grente (*22*) found that the majority of counties examined in France had no significant change in livestock predation following public wolf hunting, but a few counties experienced either increases or decreases in predation. In contrast, some studies in the United States found that targeted removal or hunting of wolves in high-risk areas can reduce subsequent livestock depredations in those specific areas (*19*, *23*–*25*). These sparse and equivocal results warrant more robust analyses to make stronger causal inferences on the impacts of wolf hunting.

Here, we investigate the effects of legalized wolf hunting on two aspects of human-wolf interactions: (i) livestock depredation by wolves and (ii) the lethal removal of wolves by government agencies from 2005 to 2021 in four states in the northwestern US—Idaho, Montana, Oregon, and Washington. For simplicity, we use the term hunting to refer to all legal and regulated methods of public harvest or offtake including trapping but excluding lethal removal by government agencies. We focus on the northwestern US because it is a nexus for conflict over ongoing efforts to conserve wolves while mitigating risks to livestock (*26*). Following a reintroduction program in the mid-1990s, the northern Rocky Mountain wolf population has quickly grown and expanded to neighboring areas (*9*). When federal protections were first removed in 2009, wolf hunting became legal in Idaho and Montana (note that hunting was periodically interrupted during various legal challenges until 2012) (see fig. S1). Meanwhile, hunting of wolves has not been legalized on nontribal lands in Oregon and Washington, states where wolves have recolonized in recent years. Where hunting has been legalized, some counties have experienced no or low levels of wolf hunting in some years compared to higher levels in other counties or years. This substantive temporal-spatial variation in hunting levels affords us an opportunity to causally infer its effects on livestock depredations and government removals.

We use an approach similar to the logic of before/after/control/impact (BACI) studies that compares geographical units that were affected by a management intervention (wolf hunting) to those that were not, both before and after said intervention. In contrast to typical BACI studies, which model the effect of a policy change at the level of a state or country, we model the effect of hunting itself and use counties as the unit of analysis to leverage the fine-scale variation. We use multiple models to balance the inherent limitations of each modeling approach. First, we apply two difference-in-differences techniques, namely, two-way fixed-effects (TWFE) and PanelMatch models, that compare differences between counties that had or did not have wolf hunting in a given year. While PanelMatch models (*27*) only account for binary independent variables (i.e., hunting or no hunting), TWFE models can be binary or continuous to account for different levels of hunting within a county. Second, we apply dynamic panel models (DPM) (*28*), which have not been used in previous studies on the impacts of wolf hunting. These dynamic models better account for the likelihood that our dependent variables (lethal removal and livestock predations) may also influence our independent variable (wolf hunting) over time.

We test effects on lethal removal and livestock predation in the current and subsequent year for all models while controlling for time-varying environmental and social factors that may influence human-wolf encounters. The environmental factors are rough proxies for resource availability (drought levels), game species abundance (elk harvest levels), and the likelihood of encountering cattle (number of cattle). The social factors are rough proxies for cultural dimensions affecting relations with wolves, such as worldviews and livelihoods (median resident age, median resident household income, and percent of presidential vote for the Republican candidate) and the likelihood of encountering people (human population size). Reliable and comparable county-level data on wolf abundances across our study region and time period were not available. Although we cannot control for wolf abundance directly, we ran robustness checks by subsetting the data from 2012 onward, in which the numbers of wolves remained mostly stable at the state level and for which we have the full suite of social and environmental covariates [e.g., (*29*)] (see figs. S12 and S13). If we assume that this translates into stable wolf abundance at the county level, then we can rule out confounding by this variable. Moreover, we ran sensitivity analysis simulating a confounder positively correlated to both our outcomes and predictor—such as wolf abundance—to understand how this would change our results (see fig. S14). We also ran robustness checks on different measurements of livestock predation including sheep only, cattle only, and animal unit months (AUMs) as a way of standardizing the size, forage needs, and even economic value of different livestock types (*30*) while we use the sum of cattle and sheep depredation in the main analyses (see figs. S6 and S7).

## RESULTS

### Effect on livestock depredation

**Fig. 1.**
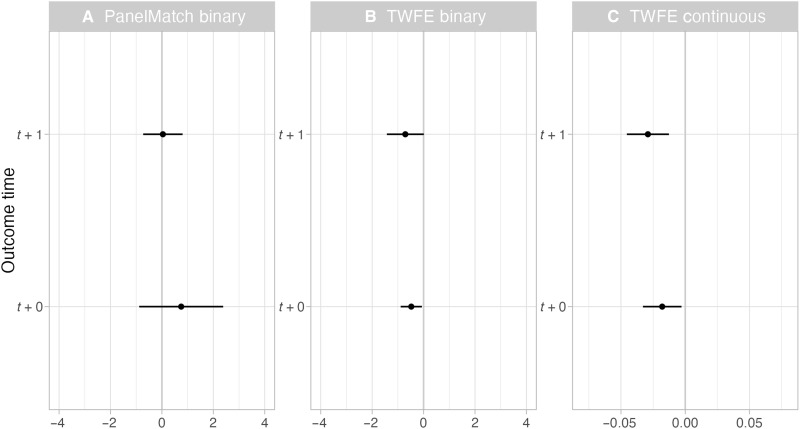
Effects of public wolf hunting on livestock predation by wolves using difference-in-differences modeling. Results of PanelMatch model where wolf hunting is binary (**A**), TWFE model where wolf hunting is binary (**B**), and TWFE model where wolf hunting is continuous (**C**). The whiskers indicate 95% confidence intervals, and where these cross zero on the *x* axis, the relationship does not significantly differ from a null effect. All model results are shown for the current year (*t* + 0) and a 1-year lag (*t* + 1). Note that the *x* axis varies across the panels.

We find consistent negative effects for the TWFE models (Fig. 1, B and C), although one of them is only marginally statistically significant. The PanelMatch model yield null effects (Fig. 1A). Collectively, these results provide some support for the idea that more hunted wolves leads to lower livestock predation—although as not all models are statistically significant, especially when examining our robustness checks, the overall picture remains mixed.

Substantively, a coefficient of −0.0179 (SE = 0.008) in the continuous TWFE model (*t* + 0) means that hunting one additional wolf is associated with a 1.77% [95% CI: 0.22%, 3.30%] decrease in expected livestock depredations. Livestock depredations averaged three per county annually, so reducing depredations by 1.77% for each wolf killed in that same year would lead to a savings of ~0.05 livestock. In a county at the 90th percentile of depredations (about five livestock depredations as baseline), those losses could be reduced by ~0.09 livestock for each wolf killed. Turning to our binary TWFE (*t* + 0), the estimated effect size of −0.479 (SE = 0.211) for hunting any wolves translates into a decrease of 38.06% [95% CI: 6.33%, 59.04%] of livestock depredations.

The seeming discrepancy between the two effect sizes stems from a highly skewed distribution of harvested wolves, with a maximum of 113 and a mean of ~5 across counties/years where hunting was legalized. Thus, the difference in the estimated effect sizes reflect different contrasts. The binary model collapses all positive values into “any hunting,” so it captures the large jump from zero to some—often non-trivial—intensity. The continuous coefficient is a per-unit effect; one unit is small relative to the observed range, so the per-unit change looks modest but compounds over realistic increments (e.g., 10 more hunted wolves translates to a predicted ~16% decrease in livestock losses; 50 more hunted wolves to a ~59% decrease). The binary estimate speaks to what happens when counties move from zero to allowing the treatment under the uptake patterns in our data—while the continuous model is more useful for planning and targeting because it maps outcomes to intensity. Accordingly, we advise to focus on effects for plausible intensity changes (e.g., per 10 additional hunted wolves) and avoid adopting the binary estimate as a stand-alone projection. The TWFE models remain largely statistically significant when measuring livestock depredation separately for sheep/cattle or in AUM (fig. S6), but not when only including counties from Montana and Idaho in the models (fig. S8). They also become insignificant when subsetting the data to 2012 and later when wolf numbers were roughly stable (fig. S12). We also simulate to what extent an unobserved confounder such as wolf abundance—positively correlated to both wolf hunting and livestock depredation—would affect our two-way fixed effects models (fig. S14). For example, if wolf abundance was correlated with both variables at *r* = 0.5, then the coefficient for the effect of wolf harvests in the continuous TWFE model (*t* + 0) would change from −0.0179 to −0.029.

**Fig. 2.**
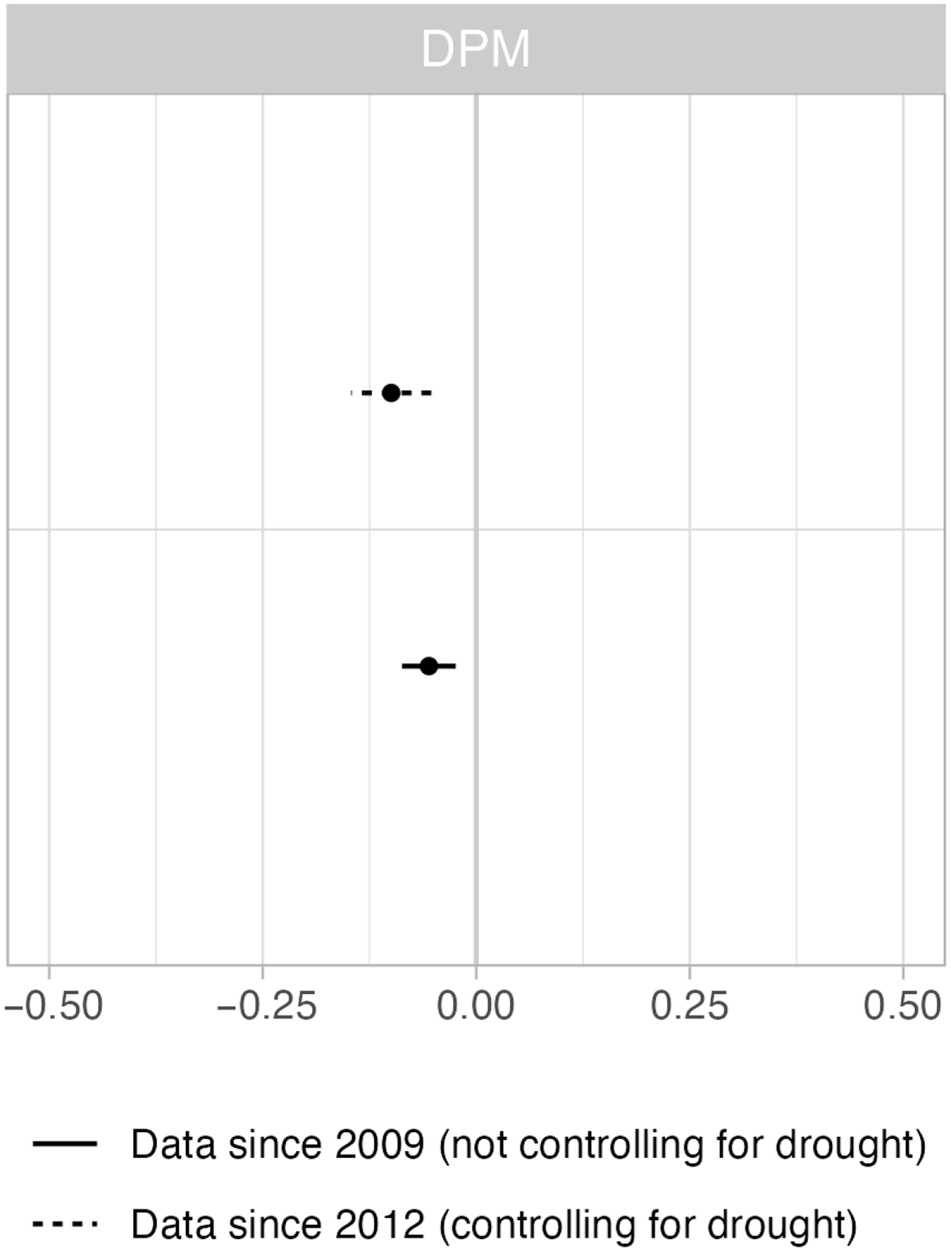
Effects of public wolf hunting on livestock predation by wolves using DPM. Results of DPM of the effects of public wolf hunting on livestock predation by wolves. We included data from 2009 to 2021 (solid line) without controlling for drought (the model did not converge due to drought indicators being constant in counties in 2011) and from 2012 to 2021 (dashed line) while controlling for drought. The whiskers indicate 95% confidence intervals, and where these cross zero on the *x* axis, the relationship does not significantly differ from a null effect.

The dynamic panel models, which allow for the possibility that public wolf hunting is affected by earlier livestock predation/government removals, indicate an effect of wolf hunting on livestock predation in both specifications (Fig. 2). We estimated a model that did not control for drought (using data from 2009 onward) and one that did control for drought (using data from 2012 onward; see Materials and Methods). Using alternative measures of predation (fig. S7) yields similar results, though not significant in all cases. Including only Montana and Idaho (fig. S9) suggests an even stronger negative effect of wolf hunting.

### Effects of wolf hunting on lethal removal of wolves

**Fig. 3.**
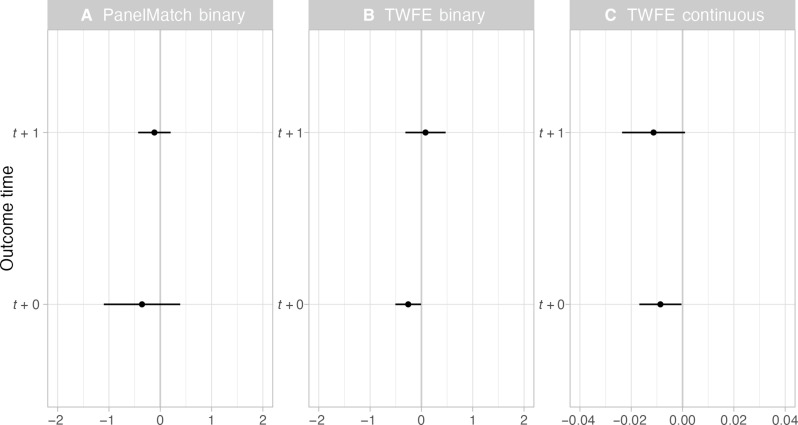
Effects of public wolf hunting on lethal removals of wolves using difference-in-differences modeling. Results of PanelMatch model where wolf hunting is binary (**A**), TWFE where wolf hunting is binary (**B**), and TWFE model where wolf hunting is continuous (**C**). The whiskers indicate 95% confidence intervals, and where these cross zero on the *x* axis, relationship does not significantly differ from a null effect. All model results are shown for the current year (*t* + 0) and a 1-year lag (*t* + 1). Note the x-axis varies across the panels.

We found no significant evidence among any of our model specifications that wolf hunts replaced the need for government agencies to lethally remove wolves (Fig. 3). Results remained substantively unchanged when subsetting the data to Montana and Idaho (fig. S10) or to 2012 and after (fig. S13).

**Fig. 4.**
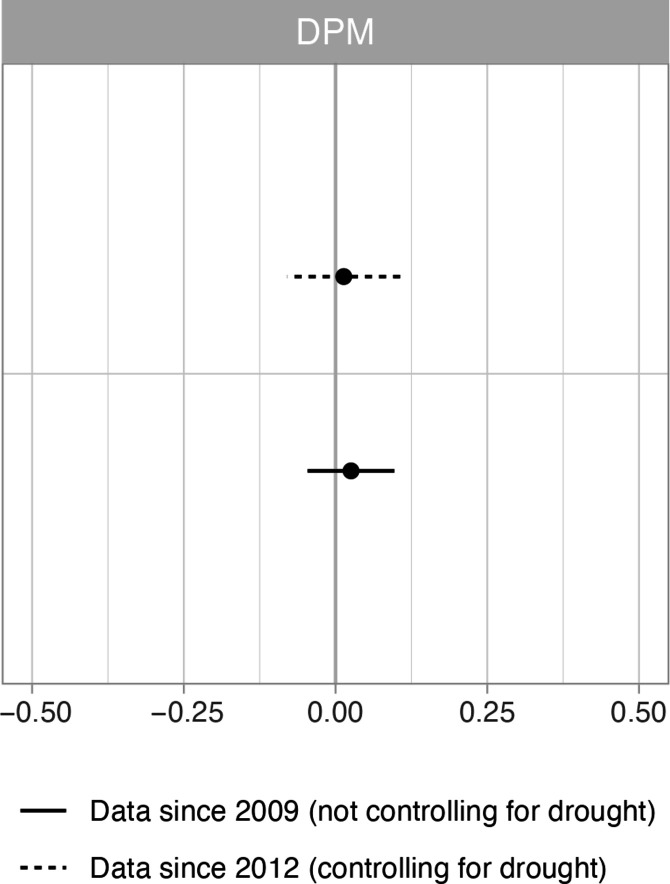
Effects of public wolf hunting on lethal removals of wolves using DPM. Results of DPM of the effects of public wolf hunting on the lethal removal of wolves by government agencies. We include data from 2009 to 2021 (solid line) without controlling for drought (the model did not converge due to drought indicators being constant in counties in 2011) and from 2012 to 2021 (dashed line) while controlling for drought. The whiskers indicate 95% confidence intervals, and where these cross zero on the *x* axis, relationship does not significantly differ from a null effect.

The dynamic panel modeling outputs (Fig. 4) also show a robust pattern of null results. We reran all these models, subsetting the data to Idaho and Montana only, with similar null results (fig. S11).

## DISCUSSION

Researchers wishing to estimate the effect of wolf hunting on livestock predations face a number of analytical challenges, from determining the appropriate scale of analysis to the selection of appropriate statistical models. We applied a variety of modeling techniques to compensate for inherent limitations of each model type and found that most but not all of our models indicate that wolf hunting reduced livestock depredations by wolves in the same or subsequent year. In contrast, none of our models indicates an effect of wolf hunting on the lethal removal of wolves by government agencies.

Although our results indicate that hunting wolves had a statistically significant effect on livestock depredations in our study area, from a practical standpoint, the size of the effect is quite small. Similarly, DeCesare *et al.* (*19*) found that wolf hunting in Montana reduced the rate of recurring livestock predation in studied areas by a modest effect size. These small effects, combined with other research showing null effects, suggest that a high percentage of wolves must be hunted before a substantive reduction in livestock predation is observed (*19*, *20*, *31*). If the threshold is very high, then it may not be feasible or desirable (socially, politically, or ecologically) to reach the threshold required to substantively impact livestock predation.

Some insight into the small effect might be provided by considering plausible mechanisms underpinning the relationship between wolf hunting and livestock predation. One mechanism is that lowering the population of wolves decreases the probability of wolves encountering livestock. DeCesare *et al.* (*19*), for example, showed an association between wolf density and depredations. This mechanism treats all wolves in the same manner; that is, it assumes a fixed encounter rate and fixed tendency of each wolf to kill livestock such that a reduction in wolves would lead to fewer encounters and, thus, fewer depredations. Alternatively, treating wolves as individuals, one might propose that hunting would reduce depredations in cases where more “bold” wolves (i.e., animals with a greater propensity than shy individuals to depredate) were removed. Relatedly, the hunting of wolves could cause these animals to behaviorally avoid people and thus make them less likely to attack livestock, at least in human-occupied areas (*25*, *31*). The effect of these mechanisms, however, may be offset if the killing of wolves disrupts social pack dynamics or age structures in ways that exacerbate livestock depredations (*32*).

Unfortunately, the data used in these analyses do not allow us to tease out which mechanisms are responsible for the reduction in depredations. Also, the myriad—likely simultaneously interacting—mechanisms generate considerable uncertainty about which mechanisms prevail under various conditions. As a result, the effects of wolf hunting on livestock depredation witnessed in our study may be unreliable or inconsistent when extended to other regions outside our study system. This uncertainty is compounded by a number of contextual factors that can vary widely, such as whether livestock producers are using nonlethal deterrents such as range riding, fladry, or removing livestock carcasses from the landscape to avoid attracting predators. Likewise, the effects may be moderated by fluctuations in key resources, such as the availability of wild prey or annual differences in weather, which can affect prey vulnerability (*33*). In our analysis, drought diminished the effect of wolf hunting on livestock depredation (Fig. 2), suggesting that water stress in the landscape altered the interaction patterns of wolves and livestock.

Unlike livestock depredations, we found no evidence that wolf hunting reduces subsequent lethal removal of wolves by government agencies. This null effect may have to do with the different spatial and temporal scales at which these management interventions operate. Public hunting is a nontargeted, population-level intervention; one that happens months after wolves typically predate on grazing livestock. In contrast to large-scale, state-wide hunting that seeks to reduce depredations by reducing wolf abundance/density, the targeted lethal removal of wolves/wolf packs by government agencies focuses on the scale at which depredations occur, removing known (or suspected individuals). Hence, a number of studies have shown that the targeted removal of specific depredating wolves may reduce future depredations (*23*, *24*), but this is likely to occur only at a very localized (e.g., pack territory) scale (*32*, *33*).

Regardless, the null effect on government removals challenges another potential use of wolf hunting, which is to reduce the need for targeted removal of wolves by government agencies. Certainly, there are social, economic, and ecological reasons why lethal removals should be minimized to the extent possible. For example, lethal removals can be very costly (*34*); Coleman (*13*) estimates the average cost to lethally remove a single wolf in Idaho was $9617. As government agencies are largely funded by taxpayer money, there is constant pressure to be economically effective. Our results suggest that legalizing wolf hunting may not address the concern over the taxpayer cost of wolf management.

Assessing the effectiveness of wolf hunting is inherently challenging for multiple reasons including the difficulty obtaining comparable data for multiple states. Key wolf data are often not publicly available or accessible in the United States (*35*, *36*). We had to submit multiple Freedom of Information Act requests and apply months of follow-up pressure to obtain some of the information in this study. Even with access, the data may not be comparable as each state collects and manages data separately after wolves were removed from federal protection. In our study region, each state uses different methods of estimating wolf population size that are not comparable and potentially unreliable within a state (*37*). While we expect hunting to affect wolf population dynamics in potentially complex ways (*38*–*40*), we were unable to account for differences in wolf population size, given the lack of reliable and comparable data. However, wolf populations in Idaho and Montana have been relatively stable at the state level since 2012; hence, we ran robustness checks that subset the data to 2012 to 2021. We included models subsetted to the stable period of 2012 to 2021 and a sensitivity analysis to address the potential impact of wolf abundance on our results.

Our study only assesses two potential benefits of wolf hunting—decreases in the number of livestock depredations and lethal removals of wolves by government agencies. We recognize that there are other justifications for the legalization of wolf hunting including increasing human tolerance for wolves, increasing wild ungulate populations, and reducing threats to human safety (*16*). Some contend that designating wildlife as a game species can, in some situations, increase the real or perceived value of the species (*41*), although it is unclear whether this is true with wolves. The United States has a long history of using hunting to manage wildlife populations, and income generated from hunting licenses helps fund efforts to mitigate negative human-wildlife interactions (*42*). Wolf hunting may be the reason for stable wolf populations, at least in parts of Idaho and Montana [see (*40*)]. Therefore, without public hunting, it is possible that wolf populations would increase and that livestock depredations and lethal removal instances would increase in turn.

There are tangible trade-offs, however, in conducting wolf hunts that are rarely addressed. For example, there are operational costs to conducting a state-wide hunting program, and it is unclear what those costs are on a per-wolf basis. Public hunting of wolves also can affect ecotourism, such as occurred when large numbers of Yellowstone wolves were killed by hunters just outside the national park boundary (*43*). Recent research has also shown that wolves reduce costs associated with deer-vehicle collisions—a savings that outweighed the financial losses from livestock predation (*44*). Thus, future work that makes a fuller, empirical appraisal of the costs and benefits from wolf hunting would better inform decision makers and the public about the trade-offs associated with this management intervention.

Even with public wolf hunting and lethal removal by government agencies in Montana and Idaho, livestock depredations remain a major concern. Effective methods of mitigating negative interactions between ranchers and wolves are urgently needed, yet the social, economic, and ecological costs and benefits need to be carefully considered. Nonlethal deterrents have been effective in some contexts (*35*, *45*, *46*), yet public funding for nonlethal deterrents is inconsistent (*47*). Changes to livestock husbandry and targeted removals by the government or ranchers are also potentially effective solutions (*19*, *23*). Ideal solutions to mitigate wolf impacts on humans are likely to vary across time and space (*46*). Ultimately, more empirical evidence is needed to determine effective and appropriate mitigation strategies that are context-specific and to design public policy accordingly (*35*). Furthermore, it is unrealistic to expect the elimination of livestock predation with wolves present. People must accept some level of risk when living in proximity to wolves. Predator compensation schemes or insurance programs have proven effective in some cases (*45*), and US communities show willingness to financially support similar programs (*46*), yet many existing insurance programs in the United States have had low rancher satisfaction (*47*).

The dearth of information on the effects of wolf management strategies hampers the ability of wildlife agencies to develop appropriate policies to support human-wolf coexistence. We used multiple modeling approaches that compare individual counties before and after hunting was legalized and compare counties with and without hunting to ensure a robust analysis of the impacts of wolf hunting on livestock depredation by wolves and lethal removal of wolves by government agencies. Our results inform the current debate surrounding the potential use of public wolf hunting as a management strategy in Europe and the United States. The minimal effects of wolf hunting on negative human-wolf interactions suggests that caution be used when implementing wildlife management strategies based on untested assumptions or claims. Instead, we urge the rigorous testing of assumptions and interventions, especially related to contentious issues such as wolf management. Adopting comparable methods of data collection and increasing the transparency and accessibility of data can facilitate testing of assumptions and interventions.

## MATERIALS AND METHODS

### Variables

We obtained temporal-spatial data on wolf hunting, wolf removals, and livestock depredation for the US states Montana, Idaho, Oregon, and Washington from 2005 to 2021. Livestock depredation data was unavailable for Montana from 2005 to 2007 and therefore was entered as null values. All our variables were measured at the county-year level. While Montana and Idaho legalized public wolf hunting during the study period, public wolf hunting was illegal in Washington and Oregon throughout the whole period (with the exception of some tribal lands). As the counties in these two states were never “treated,” we also ran our models without these two states as a robustness check.

**Fig. 5.**
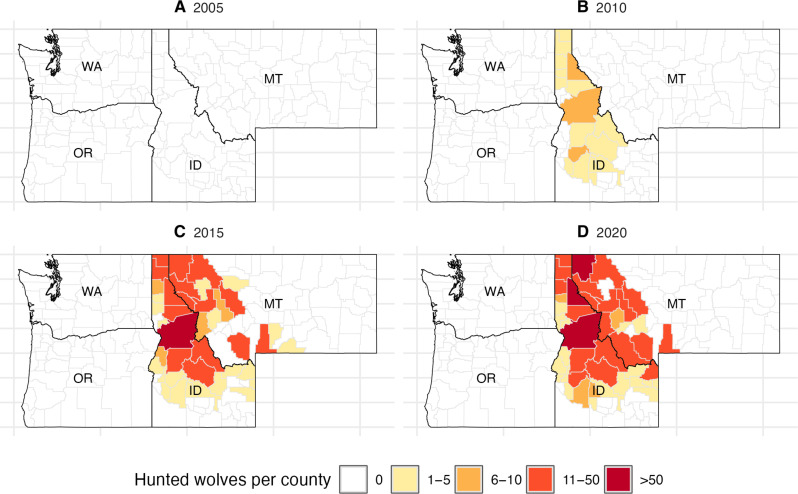
Geographic distribution of wolf hunting. We use four select time periods, 2005 (**A**), 2010 (**B**), 2015 (**C**), and 2020 (**D**) to highlight temporal changes in the number of wolves hunted or trapped at the county level in Washington (WA), Oregon (OR), Idaho (ID), and Montana (MT). White indicates zero wolves hunted or trapped, and darker shades of orange indicate higher numbers of wolves hunted or trapped.

Our independent variable is the total number of wolves hunted (i.e., the sum of legally trapped and hunted wolves) and a binary version of this variable (i.e., whether any wolves were hunted). For Idaho, we obtained the raw numbers at the level of game management units, which we converted to the county level based on the proportion of the game management unit in each county. Montana records wolves hunted at the county level. The number of counties with wolf hunting and the number of wolves hunted in a county generally increased over time for Montana and Idaho (Fig. 5 and figs. S1 and S2).

**Fig. 6.**
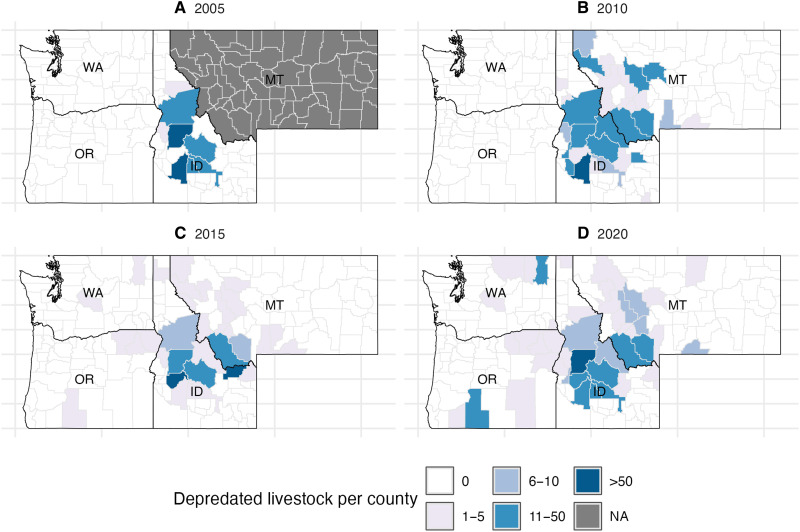
Geographic distribution of livestock predated. We use four select time periods, 2005 (**A**), 2010 (**B**), 2015 (**C**), and 2020 (**D**) to highlight temporal changes in the number of livestock predated by wolves at the county level in Washington, Oregon, Idaho, and Montana. White indicates zero lethal removals and darker shades of blue indicate higher numbers of livestock predated. Predation data was unavailable in Montana for 2005 to 2007 and was therefore entered as null values.

**Fig. 7.**
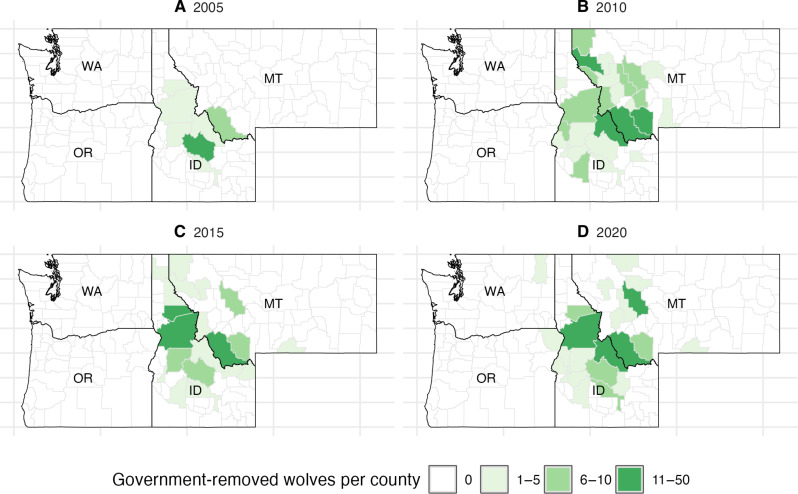
Geographic distribution of lethal removal of wolves by government agencies. We use four select time periods, 2005 (**A**), 2010 (**B**), 2015 (**C**), and 2020 (**D**) to highlight temporal changes in the number of wolves lethally removed by government agencies at the county level in Washington, Oregon, Idaho, and Montana. White indicates zero lethal removals, and darker shades of green indicate higher numbers of lethal removals.

Our first dependent variable was the total number of livestock predated by wolves according to reports from state agencies. For our main analysis, we use the sum of cattle and sheep that were confirmed or probable cases of wolf depredation. Cattle and sheep account for the majority of wolf depredations. For robustness analyses, we also model the effect on cattle and sheep depredation separately, and we include AUM as a measure of depredation to account for the size, forage requirements, and even economic value of cows/sheep. When applying an AUM approach, we multiply the number of sheep predated by 0.2 and cows by 0.92 without differentiating adults/juveniles as that information is not consistently available in the records (*30*). Figure 6 illustrates the geographic variation of livestock depredation over time (see fig. S3 for all years). Our second outcome is the total number of wolf lethal removals by government agencies [obtained from US Department of Agriculture Animal and Plant Health Inspection Service (USDA APHIS) and state wildlife agency reports], displayed in Fig. 7 (see fig. S4 for all years).

### Covariates

We further controlled for a number of time-variant variables that could confound the relationship between public wolf harvests and our outcomes. First, drought could influence abundance of wolves and therefore both their predation behavior and opportunity to hunt them. We obtained a measure of drought (recoded to an index from 0 = no drought to 5 = severe drought) from the US Drought Monitor (*48*). Second, we controlled for the number of elk harvested in a county as an indicator for game abundance, which could both influence behavior of wolf hunters/trappers and predation behavior of wolves. Elk harvest data at the game management unit scale was reassigned to county scale according to the proportion of the game management unit in each county. Third, an increase (decrease) in cattle could result in more (less) opportunity for livestock depredation and also greater support for wolf harvesting. Data on cattle numbers were obtained from the National Agricultural Statistics Service (*49*). Because political affiliation has been linked to wolf-related attitudes and behavior (*50*, *51*), we controlled for the percentage of Republican votes for the Presidential candidate using MIT Election Lab data (*52*). Last, we controlled for potential changes in a county’s demographic structure that could affect both livestock ranching and wolf hunting practices. Specifically, we used county-level population size, median household income, and median age, all collected from the US Census American Community Survey (*53*). As some of our modeling approaches do not handle extremely skewed independent variables well, we log-transformed the number of cattle and the number of harvested elk.

### Statistical analysis

To estimate the effect of public wolf harvest on livestock depredation, we take advantage of the within-county variation in the independent variable, i.e., wolves hunted, over time. That is, our models test whether levels of wolf hunting that are higher (lower) for a county, compared to itself, are associated with higher (lower) levels in the outcome. We follow two slightly different analytical approaches: first, difference-in-differences panel modeling, and second, dynamic panel modeling (a form of structural equation modeling).

#### Difference-in-differences panel modeling

First, we consider our dataset as a case for difference-in-differences panel modeling. Conceptually, this approach compares the difference in the outcome of units before and after being “treated” (i.e., counties in which legal wolf hunting occurred) to the difference in the outcome between the same time points for control units (i.e., counties in which wolf hunting never happened). This allows one to disregard any time-invariant differences between counties (such as the percent of federal land) and time-variant factors that affect all counties equally (such as changing climate). The main threat to identify causality is the existence of unmeasured time-variant, unit-variant confounders.

The classic technique to pursue this strategy is the TWFE model. In our case, this model regresses the outcome *y*_it_ (i.e., livestock depredation or government removals) on an indicator for the geographic unit α_i_ (i.e., county), an indicator for the time period μ_t_ (i.e., year), the independent variable *x*_it_ (i.e., wolves hunted, either as continuous or binary variable), a vector of controls *Z*_it_ (i.e., drought level, elk harvests, number of cattle, population size, median age, and median household income), and the outcome level of the previous year *y*_it−1_. As both outcomes are an overdispersed, non-negative variable, we use a negative binomial model. Formally, it can be written as follows, with β_1_ being the coefficient of interest

$$y_{\text{it}}∼\text{NB}\left(\mu ,k\right)$$

$$\mu_{\text{it}}=\text{exp}\left(\alpha_{i}+\mu_{t}+\beta_{1}x_{\text{it}}+\beta_{2}y_{\text{it}-1}+\beta_{3}Z_{\text{it}}+\epsilon_{\text{it}}\right)$$

TWFE models have been found to be potentially biased when the treatment occurs at different time points across units and units move in and out of treatment (*54*), which is the case in our data. Hence, we alternatively apply the recently developed PanelMatch algorithm (*27*). Briefly, the algorithm matches counties that receive treatment (i.e., wolves hunted) after previously being untreated to control counties (i.e., no wolves hunted) with identical treatment histories, making the matched sets as similar as possible using a set of covariates (i.e., drought level, elk harvests, number of cattle, population size, median age, median household income, and presidential vote). As a refinement method, we use the Mahalanobis distance with a match set of 10 and a lag of eight periods. The approach then computes a difference-in-differences estimate by comparing treated units to their matched controls.

In contrast to TWFE, PanelMatch does not allow for a continuous treatment. As the number of wolves hunted is continuous, both approaches therefore have their strengths and weaknesses, and we present results from both. Across both models, we run two versions of all our models: one modeling the effect of wolf harvests on the outcomes in the same year, and another modeling the effect on the outcomes in the subsequent year.

#### Dynamic panel modeling

The problem of classic difference-in-differences-style panel modeling is that it does not allow for the independent variable of one period to be influenced by the dependent variable of an earlier period. This is likely in our case: Although we are interested in whether public wolf hunting reduces livestock depredation, it is likely that the level of livestock depredation in a county influences the number of hunted wolves, as wolf hunting is partly a reaction to livestock depredation. Structural equation modeling (SEM), particularly the tradition of cross-lagged panel modeling, accommodates for such two-directional causalities. Hence, as a second strategy, we estimate the “dynamic panel model” (*28*). The estimation method proposed by Allison *et al.* (*28*) also performs well for highly skewed distributions.

Formally, the model can be portrayed by the two following equations. In the first equation, *y*_it_ represents the outcome; α_i_ represents the effect on the outcome of all unobserved, time-constant variables (i.e., the county fixed effect); μ_t_ represents the effect of unobserved factors that vary over time but are constant across units (i.e., the year fixed effect); and *Z*_it_ represents the vector of controls. The outcome is regressed on the lagged version of both the outcome, *y*_it−1_, and the predictor, *x*_it−1_, with the coefficient of interest being β_1_

$$y_{\text{it}}=\alpha_{i}+\mu t+\beta_{1}x_{\mathrm{it}-1}+\beta_{2}y_{\mathrm{it}-1}+\beta_{3}Z_{\mathrm{it}}+\epsilon_{\text{it}}$$

The key ingredient of SEM is to simultaneously estimate a second equation shown below, which regresses the predictor on its lagged version *x*_it−1_, on the lagged outcome *y*_it−1_ and again a county effect η_i_, a year effect τ_t_, and a vector of controls *Z*_it_. In contrast to other recently developed cross-lagged models [e.g., the random-intercept cross-lagged model by (*55*)], the dynamic model specifies the unit effects α_i_ and η_i_ as “fixed,” which makes it as comparable as possible to our TWFE and PanelMatch specifications.

$$x_{\text{it}}=\eta_{i}+\tau_{t}+\gamma_{1}x_{\text{it}-1}+\gamma_{2}y_{\text{it}-1}+\gamma_{3}Z_{\text{it}}+\epsilon_{\text{it}}$$

**Fig. 8.**
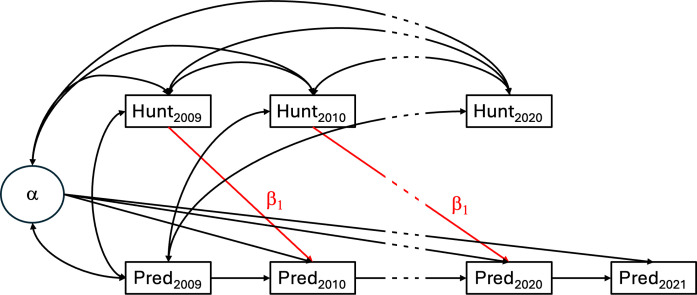
Path diagram of DPM testing the effect of wolf hunting (“hunt”) on livestock depredation (“pred”). The paths of interest are highlighted in red, and for simplicity, we exclude paths from 2011 to 2019 as indicated by the dashed lines.

Same as all structural equation models, the dynamic panel model can be illustrated with a path diagram, which we do in Fig. 8. For easier illustration, we only show the model testing effects on livestock depredation and omit covariates from the graph. We also only show the first and last years (intermediate time period indicated by dashed lines).

Note that because age, population, and income vary little over time, some of the dynamic models do not converge when including them as controls, so we do not include them in Z_it_. We run two different specifications of the above model. First, as drought is constant across counties in 2011 and thus the model does not converge, we run one version using data from 2012 onwards, controlling for elk harvests and drought levels. Second, we estimate a model using data from 2009 onward, but not controlling for drought. The data are restricted to post-2009 because the SEM framework only works with data that varies across counties (i.e., there must be counties with wolf hunting and without wolf hunting). In both specifications, we use full information maximum likelihood to account for missing data and apply maximum likelihood estimation with robust (Huber-White) SEs. We allowed the coefficients of both predictor and outcome to vary over time period and reported a weighted average of the coefficient of the predictor.

## Supplementary Materials

### This PDF file includes:

Supplementary Text Figs. S1 to S14

Erratum published 2 January 2026:
The original version of the Research Article contained an error with the livestock predation data used in the publication. In the Supplementary Materials, figs. S3. S5, S6, S8, S9, S12, and S14 have been updated to include the corrected data. The captions for figs. S3 and S5 have been updated to include the following sentence: “Predation data was unavailable in Montana for 2005-2007 and was therefore entered as null values.” The original version of the Supplementary Materials PDF is available here:

## References

[1] A. R. Braczkowski, C. J. O’Bryan, C. Lessmann, C. Rondinini, A. P. Crysell, S. Gilbert, M. Stringer, L. Gibson, D. Biggs, The unequal burden of human-wildlife conflict. Commun. Biol. 6, 182 (2023). 36823291 10.1038/s42003-023-04493-y PMC9950466

[2] C. E. Wilkinson, A. McInturff, J. R. B. Miller, V. Yovovich, K. M. Gaynor, K. Calhoun, H. Karandikar, J. V. Martin, P. Parker-Shames, A. Shawler, A. Van Scoyoc, J. S. Brashares, An ecological framework for contextualizing carnivore–livestock conflict. Conserv. Biol. 34, 854–867 (2020). 32406970 10.1111/cobi.13469

[3] M. J. Manfredo, T. L. Teel, R. E. W. Berl, J. T. Bruskotter, S. Kitayama, Social value shift in favour of biodiversity conservation in the United States. Nat. Sustain. 4, 323–330 (2021).

[4] R. Niemiec, R. E. W. Berl, M. Gonzalez, T. Teel, C. Camara, M. Collins, J. Salerno, K. Crooks, C. Schultz, S. Breck, D. Hoag, Public perspectives and media reporting of wolf reintroduction in Colorado. PeerJ 8, e9074 (2020). 32435536 10.7717/peerj.9074 PMC7224228

[5] E. R. Olson, J. L. Stenglein, V. Shelley, A. R. Rissman, C. Browne-Nuñez, Z. Voyles, A. P. Wydeven, T. Van Deelen, Pendulum swings in wolf management led to conflict, illegal kills, and a legislated wolf hunt. Conserv. Lett. 8, 351–360 (2015).

[6] J. T. Bruskotter, The predator pendulum revisited: Social conflict over wolves and their management in the western United States. Wildl. Soc. Bull. 37, 674–679 (2013).

[7] T. A. Messmer, D. Reiter, B. C. West, Enhancing wildlife sciences’ linkage to public policy: Lessons from the predator-control pendulum. Wildl. Soc. Bull. 29, 1253–1259 (2001).

[8] J. T. Bruskotter, J. A. Vucetich, M. J. Manfredo, G. R. Karns, C. Wolf, K. Ard, N. H. Carter, J. V. López-Bao, G. Chapron, S. D. Gehrt, W. J. Ripple, Modernization, risk, and conservation of the World’s largest carnivores. Bioscience 67, 646–655 (2017).

[9] L. D. Mech, Where can wolves live and how can we live with them? Biol. Conserv. 210, 310–317 (2017).

[10] D. E. Ausband, L. D. Mech, The challenges of success: Future wolf conservation and management in the United States. Bioscience 73, 587–591 (2023).

[11] L. O’Carroll, “EU to rethink conservation status of wolves after numbers surge,” *The Guardian*, 4 September 2023; www.theguardian.com/environment/2023/sep/04/eu-to-rethink-conservation-status-of-wolves-after-numbers-surge.

[12] IDFG, Idaho Gray Wolf Management Plan 2023–2028 (2023); https://idfg.idaho.gov/sites/default/files/idaho-gray-wolf-management-plan-2023-2028.pdf.

[13] N. M. Coleman, “Comparing the Economic Costs of Lethal and Non-Lethal Control of Wolf Depredation on Sheep in Southern Idaho,” thesis, Michigan Technological University, MI, USA (2023).

[14] C. T. Darimont, H. Hall, L. Eckert, I. Mihalik, K. Artelle, A. Treves, P. C. Paquet, Large carnivore hunting and the social license to hunt. Conserv. Biol. 35, 1111–1119 (2021). 33047399 10.1111/cobi.13657 PMC8359201

[15] J. Vucetich, M. P. Nelson, *Wolf hunting and the ethics of predator control* (The Oxford handbook of animal studies, 2014), pp. 411–429.

[16] A. Treves, L. M. Elbroch, J. Bruskotter, “Evaluating Fact Claims Accompanying Policies to Liberalize the Killing of Wolves,” in *Wildlife Conservation & Management in the 21st Century Issues, Solutions, and New Concepts*, G. Proulx (Alpha Wildlife Publications, 2023).

[17] J. A. Vucetich, J. T. Bruskotter, M. P. Nelson, R. O. Peterson, J. K. Bump, Evaluating the principles of wildlife conservation: A case study of wolf (*Canis lupus*) hunting in Michigan, United States. J. Mammal. 98, 53–64 (2017).

[18] G. Tomma, Europe divided on proposal to allow wolf culls. Science 383, 355 (2024). 38271511 10.1126/science.ado2399

[19] N. J. DeCesare, S. M. Wilson, E. H. Bradley, J. A. Gude, R. M. Inman, N. J. Lance, K. Laudon, A. A. Nelson, M. S. Ross, T. D. Smucker, Wolf-livestock conflict and the effects of wolf management. J. Wildl. Manag. 82, 711–722 (2018).

[20] M. Krofel, R. Černe, K. Jerina, Effectivness of wolf (*Canis lupus*) culling as a measure to reduce livestock depredations (Biotechnical Faculty, University of Ljubljana and the Slovenian Forestry Institute, 2011), pp. 11–21.

[21] M. Kutal, M. Duľa, A. R. Selivanova, J. V. López-Bao, Testing a conservation compromise: No evidence that public wolf hunting in Slovakia reduced livestock losses. Conserv. Lett. 17, e12994 (2024).

[22] O. Grente, résentation des objectifs et de la méthodologie de la these su r l’efficacité des tirs de loup et la gestion adaptative du loup, menée conjointement pa r l’ONCFS et le CEFE, thesis, Gieres, France (2021).

[23] E. H. Bradley, H. S. Robinson, E. E. Bangs, K. Kunkel, M. D. Jimenez, J. A. Gude, T. Grimm, Effects of wolf removal on livestock depredation recurrence and wolf recovery in Montana, Idaho, and Wyoming. J. Wildl. Manage. 79, 1337–1346 (2015).

[24] N. Poudyal, N. Baral, S. T. Asah, Wolf lethal control and livestock depredations: Counter-evidence from respecified models. PLOS ONE 11, e0148743 (2016). 26866592 10.1371/journal.pone.0148743 PMC4751083

[25] E. K. Harper, W. J. Paul, L. D. Mech, S. Weisberg, Effectiveness of lethal, directed wolf-depredation control in Minnesota. J. Wildl. Manage. 72, 778–784 (2008).

[26] J. T. Bruskotter, S. A. Enzler, A. Treves, Science and law. Rescuing wolves from politics: Wildlife as a public trust resource. Science 333, 1828–1829 (2011). 21960614 10.1126/science.1207803

[27] K. Imai, I. S. Kim, E. H. Wang, Matching methods for causal inference with time-series cross-sectional data. Am. J. Polit. Sci. 67, 587–605 (2023).

[28] P. D. Allison, R. Williams, E. Moral-Benito, Maximum likelihood for cross-lagged panel models with fixed effects. Socius 3, 237802311771057 (2017).

[29] S. Sells, A. Keever, M. Mitchell, J. Gude, K. Podruzny, R. Inman, Improving Estimation of Wolf Recruitment and Abundance, and Development of an Adaptive Harvest Management Program for Wolves in Montana (Montana Fish, Wildlife and Parks, 2020).

[30] K. Launchbaugh, Forage production and carrying capacity: Guidelines for setting a proper stocking rate. (University of Idaho, 2014).

[31] R. M. Anderson, S. Charnley, K. Epstein, K. M. Gaynor, J. V. Martin, A. McInturff, The socioecology of fear: A critical geographical consideration of human-wolf-livestock conflict. Can. Geogr. Géogr. Can. 67, 17–34 (2023).

[32] K. A. Cassidy, B. L. Borg, K. J. Klauder, M. S. Sorum, R. Thomas-Kuzilik, S. R. Dewey, J. A. Stephenson, D. R. Stahler, T. D. Gable, J. K. Bump, A. T. Homkes, S. K. Windels, D. W. Smith, Human-caused mortality triggers pack instability in gray wolves. Front. Ecol. Environ. 21, 356–362 (2023).

[33] L. D. Mech, D. W. Smith, K. M. Murphy, D. R. MacNulty, Winter severity and wolf predation on a formerly wolf-free elk herd. J. Wildl. Manage. 65, 998–1003 (2001).

[34] C. Lorand, A. Robert, A. Gastineau, J.-B. Mihoub, C. Bessa-Gomes, Effectiveness of interventions for managing human-large carnivore conflicts worldwide: Scare them off, don’t remove them. Sci. Total Environ. 838, 156195 (2022). 35623521 10.1016/j.scitotenv.2022.156195

[35] P. Kareiva, S. K. Attwood, K. Bean, D. Felix, M. Marvier, M. L. Miketa, E. Tate-Pulliam, A new era of wolf management demands better data and a more inclusive process. Conserv. Sci. Pract. 4, e12821 (2022).

[36] L. Merz, N. T. Bergmann, C. L. Brown, J. V. Martin, C. B. Wardropper, J. T. Bruskotter, N. H. Carter, State-level variation drives wolf management in the northwestern United States. Environ. Res. Ecol. 4, 015008 (2025).

[37] D. E. Ausband, L. N. Rich, E. M. Glenn, M. S. Mitchell, P. Zager, D. A. W. Miller, L. P. Waits, B. B. Ackerman, C. M. Mack, Monitoring gray wolf populations using multiple survey methods. J. Wildl. Manage. 78, 335–346 (2014).

[38] D. E. Ausband, Gray wolf harvest in Idaho. Wildl. Soc. Bull. 40, 500–505 (2016).

[39] J. A. Gude, M. S. Mitchell, R. E. Russell, C. A. Sime, E. E. Bangs, L. D. Mech, R. R. Ream, Wolf population dynamics in the U.S. Northern Rocky Mountains are affected by recruitment and human-caused mortality. J. Wildl. Manage. 76, 108–118 (2012).

[40] D. E. Ausband, M. S. Mitchell, L. P. Waits, Effects of breeder turnover and harvest on group composition and recruitment in a social carnivore. J. Anim. Ecol. 86, 1094–1101 (2017). 28555834 10.1111/1365-2656.12707

[41] W. L. Regelin, Wolf Management in Alaska with an Historic Perspective (Alaska Department of Fish and Game, 2002); www.adfg.alaska.gov/index.cfm?adfg=intensivemanagement.historicwolf.

[42] Association of Fish and Wildlife Agencies, Understanding the Management, Funding, and Staffing of Human-Wildlife Conflicts by State Fish and Wildlife Agencies | Wildlife Management Institute (Wildlife Management Institute, 2024); https://wildlifemanagement.institute/outdoor-news-bulletin/july-2024/understanding-management-funding-and-staffing-human-wildlife.

[43] D. W. Smith, P. J. White, D. R. Stahler, A. Wydeven, D. E. Hallac, Managing wolves in the Yellowstone area: Balancing goals across jurisdictional boundaries. Wildl. Soc. Bull. 40, 436–445 (2016).

[44] J. L. Raynor, C. A. Grainger, D. P. Parker, Wolves make roadways safer, generating large economic returns to predator conservation. Proc. Natl. Acad. Sci. U.S.A. 118, e2023251118 (2021). 34031245 10.1073/pnas.2023251118 PMC8179214

[45] J. S. Alexander, B. Agvaantseren, E. Gongor, T. N. Mijiddorj, T. Piaopiao, S. Redpath, J. Young, C. Mishra, Assessing the effectiveness of a community-based livestock insurance program. Environ. Manag. 68, 87–99 (2021). 10.1007/s00267-021-01469-8 33844062

[46] L. M. van Eeden, C. Bogezi, D. Leng, J. M. Marzluff, A. J. Wirsing, S. Rabotyagov, Public willingness to pay for gray wolf conservation that could support a rancher-led wolf-livestock coexistence program. Biol. Conserv. 260, 109226 (2021).

[47] R. Nickerson, D. Hoag, P. H. Evangelista, R. Niemiec, A. Few, S. W. Breck, U.S. livestock producer interest in alternatives to compensation programs for wolf depredation. Hum. Dimens. Wildl., 1–16 (2024).

[48] NDMC, USDA, NOAA, Drought Monitor (2000); https://droughtmonitor.unl.edu/DmData/GISData.aspx [accessed 16 May 2002].

[49] USDA, National Agricultural Statistics Service 5-year Estimates, (2022); https://quickstats.nass.usda.gov/.

[50] M. A. Ditmer, R. M. Niemiec, G. Wittemyer, K. R. Crooks, Socio-ecological drivers of public conservation voting: Restoring gray wolves to Colorado, USA. Ecol. Appl. 32, e2532 (2022). 35044025 10.1002/eap.2532

[51] L. M. van Eeden, S. S. Rabotyagov, M. Kather, C. Bogezi, A. J. Wirsing, J. Marzluff, Political affiliation predicts public attitudes toward gray wolf (*Canis lupus*) conservation and management. Conserv. Sci. Pract. 3, e387 (2021).

[52] MIT Election Data and Science Lab, County Presidential Election Returns 2000-2020}, Harvard Dataverse (2021); 10.7910/DVN/VOQCHQ.

[53] U.S. Census Bureau, American Community Survey 5-year Estimates 2006–2010, 2011–2015, 2016–2020 (2022); www.census.gov/data/developers.html.

[54] J. Roth, P. H. C. Sant’Anna, A. Bilinski, J. Poe, What’s trending in difference-in-differences? A synthesis of the recent econometrics literature. J. Econom. 235, 2218–2244 (2023).

[55] E. L. Hamaker, R. M. Kuiper, R. P. P. P. Grasman, A critique of the cross-lagged panel model. Psychol. Methods 20, 102–116 (2015). 25822208 10.1037/a0038889

