# Supplementary material for: Elusive effects of legalized wolf hunting on human-wolf interactions
Source: Sci Adv. 2025 Aug 20;11(34):eadu8945. doi: 10.1126/sciadv.adu8945 (PMC12366702; doi:10.1126/sciadv.adu8945)
Supplement: Supplementary file 2 — Erratum published 2 January 2026: The original version of the Research Article contained an error with the livestock predation data used in the publication. In the Supplementary Materials, figs. S3. S5, S6, S8, S9, S12, and S14 have been updated to include the corrected data. The captions for figs. S3 and S5 have been updated to include the following sentence: “Predation data was unavailable in Montana for 2005-2007 and was therefore entered as null values.” The original version of the Supplementary Materials PDF is available here: [file sciadv.adu8945_sm.v1.pdf]

Science Advances
AAAS

Supplementary Materials for

Elusive effects of legalized wolf hunting on human-wolf interactions

Leandra M. Merz *et al.*

Corresponding author: Leandra M. Merz, lmerz@sdsu.edu

Sci. Adv. **11**, eadu8945 (2025)
DOI: 10.1126/sciadv.adu8945

This PDF file includes:

Supplementary Text
Figs. S1 to S14

Supplementary Text

Variable descriptions

Below are additional figures to provide more details on spatial and temporal trends in the variables used for our analyses. Figure S1 provides a county-level glimpse at temporal trends in wolf hunting as a binary variable. Figures S2-4 provide spatial distribution of wolf hunting, livestock predation, and lethal removal by government agencies, respectively for each year of our study period while the figures in the main text include a smaller sample. Figure S5 shows state-level trends in the wolf hunting, livestock predation, and lethal removal over the study period. This highlights the high level of inter-annual variance in each of these variables.

Robustness checks

As reported in the main manuscript we ran multiple robustness checks on alternative measurements of livestock predation and alternative geographic/temporal ranges. Additional information and the corresponding figures are included in this section.

Alternative measurements of livestock predation

As a measurement of robustness, we repeated our analysis using different measurements of livestock predation- cattle predation only, sheep predation only, and AUM (animal unit month) equivalent calculating all sheep as 0.2 AUM and all cattle as 0.92. We did not incorporate other types of livestock as they constitute a small minority of livestock predation by wolves. Figures S6-S7 show the model results using AUM, number of cattle only, and number of sheep only.

Restricted geographical range

For our analyses we included counties in Washington and Oregon as the wolf range has expanded into these two states yet hunting is not legalized at the state level for either state. Given differences in the recolonization timeline for Washington and Oregon among other factors, we ran the analyses for counties in Montana and Idaho and the results are visualized in Figures S8-S11.

Restricted temporal range

Wolf abundance is a potential confounding variable that we cannot account for due to the unavailability of comparable wolf abundance data at the county level across our study area. Yet, wolf abundance was stable at the state level in Montana and Idaho from 2012 through 2021. Therefore, we ran our difference-in-differences modeling for a restricted temporal range of 2012 to 2021 as another robustness check and the results are visualized in Figures S12-S13.

Sensitivity analysis

To test the potential impact of a confounding variable such as wolf abundance, we conducted a sensitivity analysis for the two-way fixed effects model. We simulated an unobserved confounder that are correlated to varying degrees with both the treatment (wolves hunted) and the outcome (livestock depredation). We generated a sequence of simulated confounders with increasing correlation strength and re-estimated the models while sequentially including each confounder and the results are visualized in Figure S14.

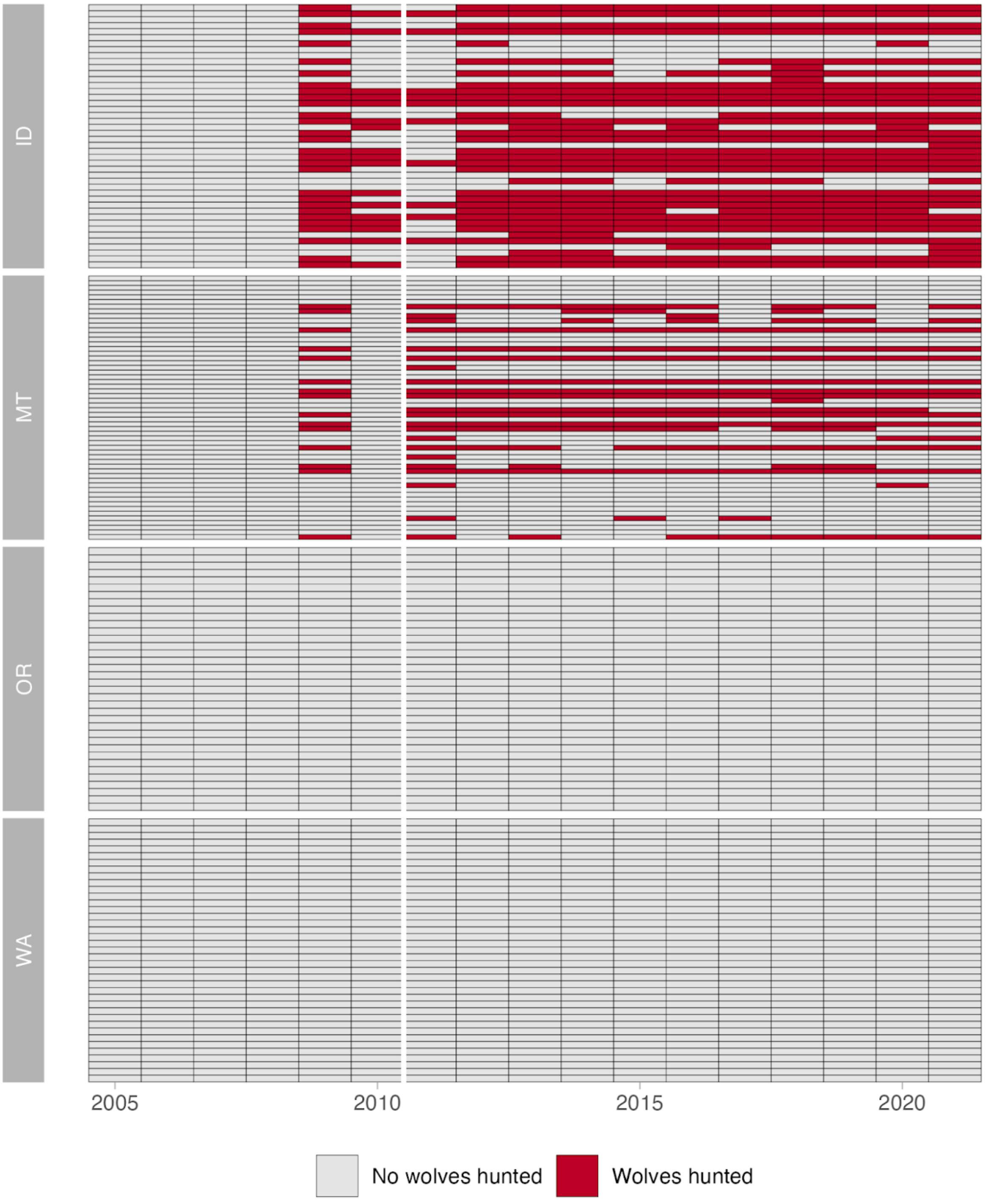

Fig. S1.

Wolf hunting treatment variation plot. Each cell represents a county in a given year. Counties are grouped by state, delineated by the horizontal white lines (cf. Figures S8 through S11 for robustness checks using Montana and Idaho data only). The vertical white line represents the cutoff for the robustness check in which we subset the data to the post-2010 period when wolf abundance was constant on the state level (cf. Figures S12 through S13).

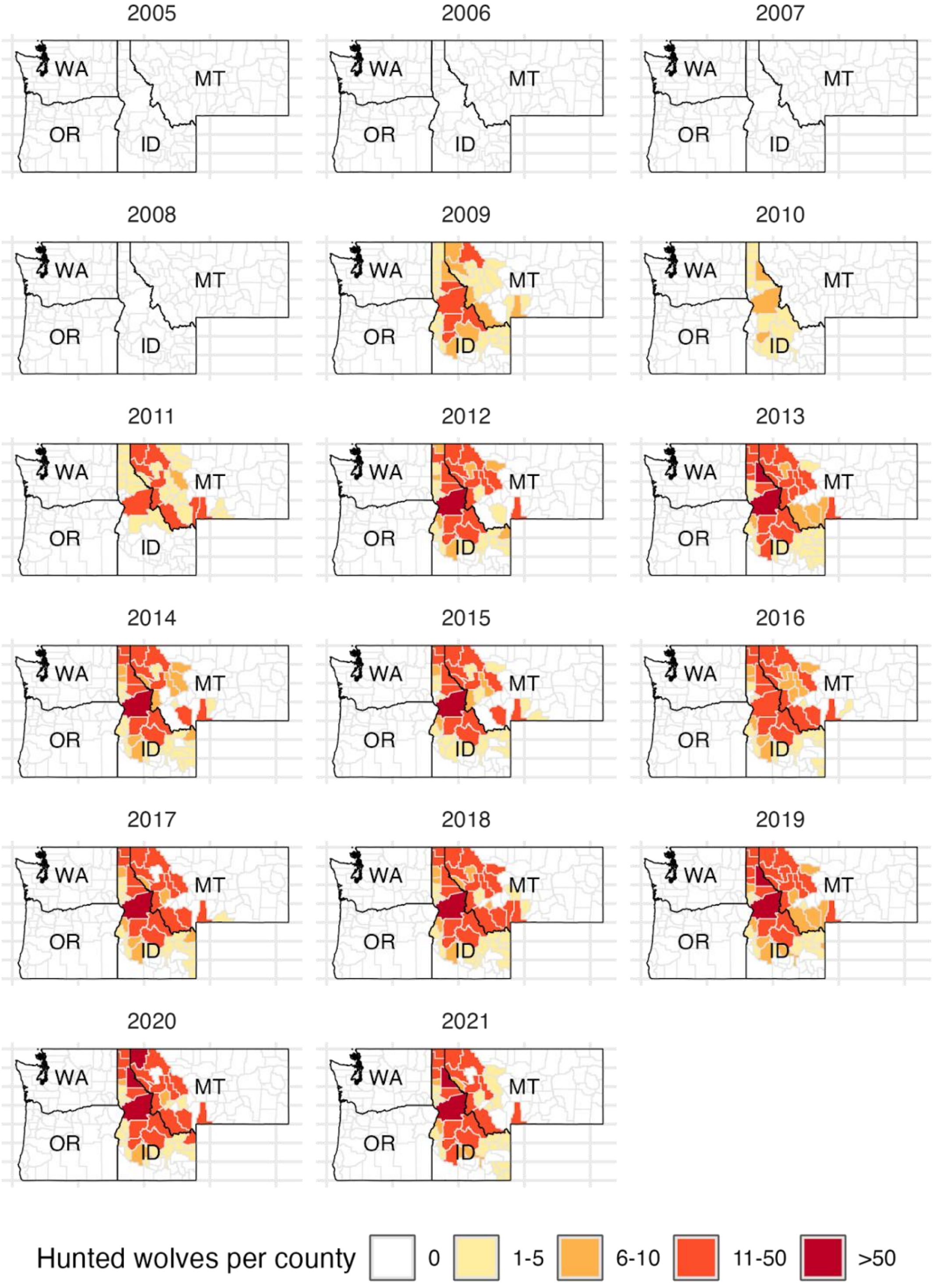

Fig. S2.

Distribution of hunted wolf across counties and over time. Darker colors represent higher numbers of hunted wolves.

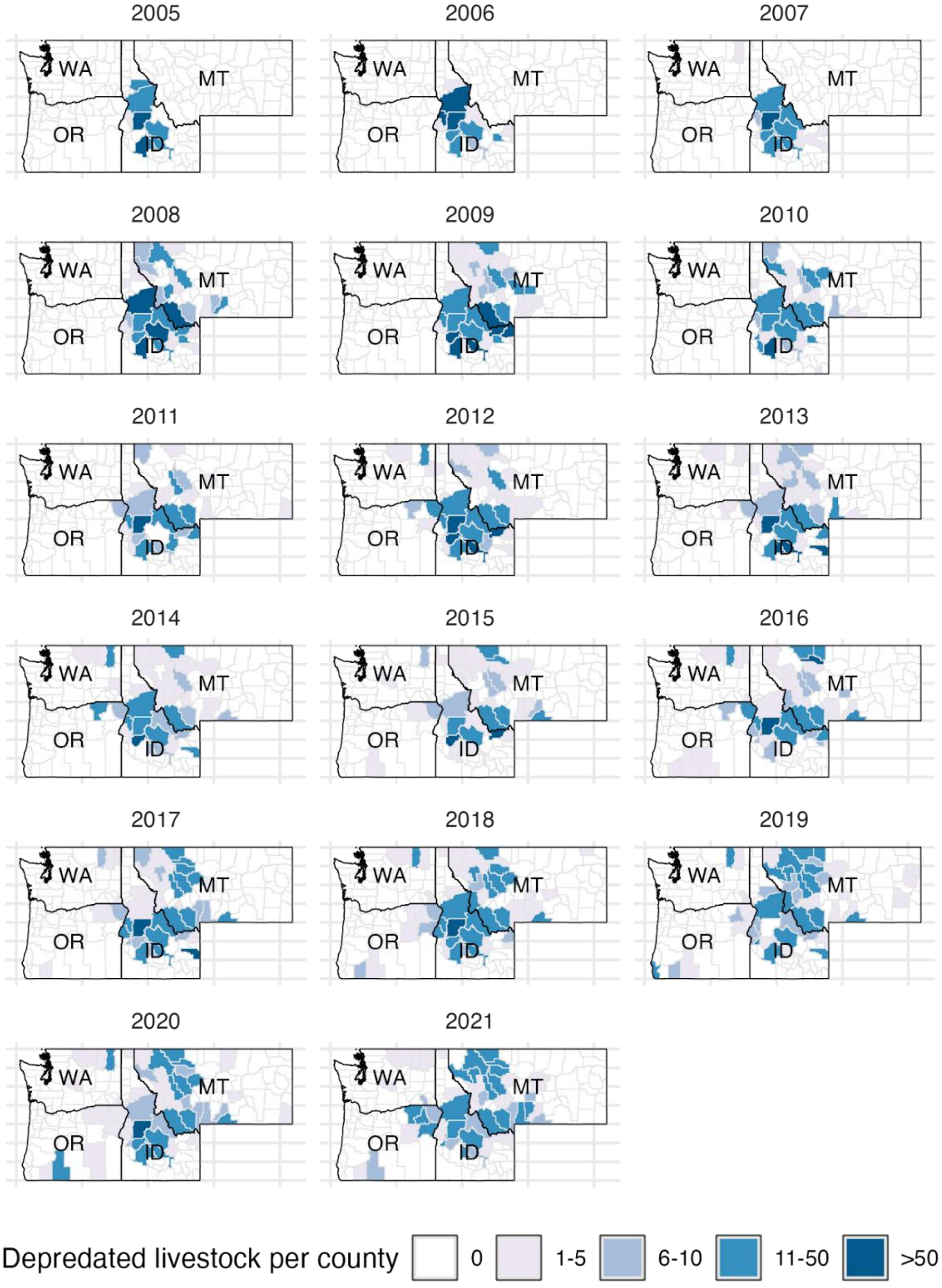

Fig. S3.

Distribution of livestock depredation across counties and over time. Darker colors represent higher numbers of livestock depredated by wolves.

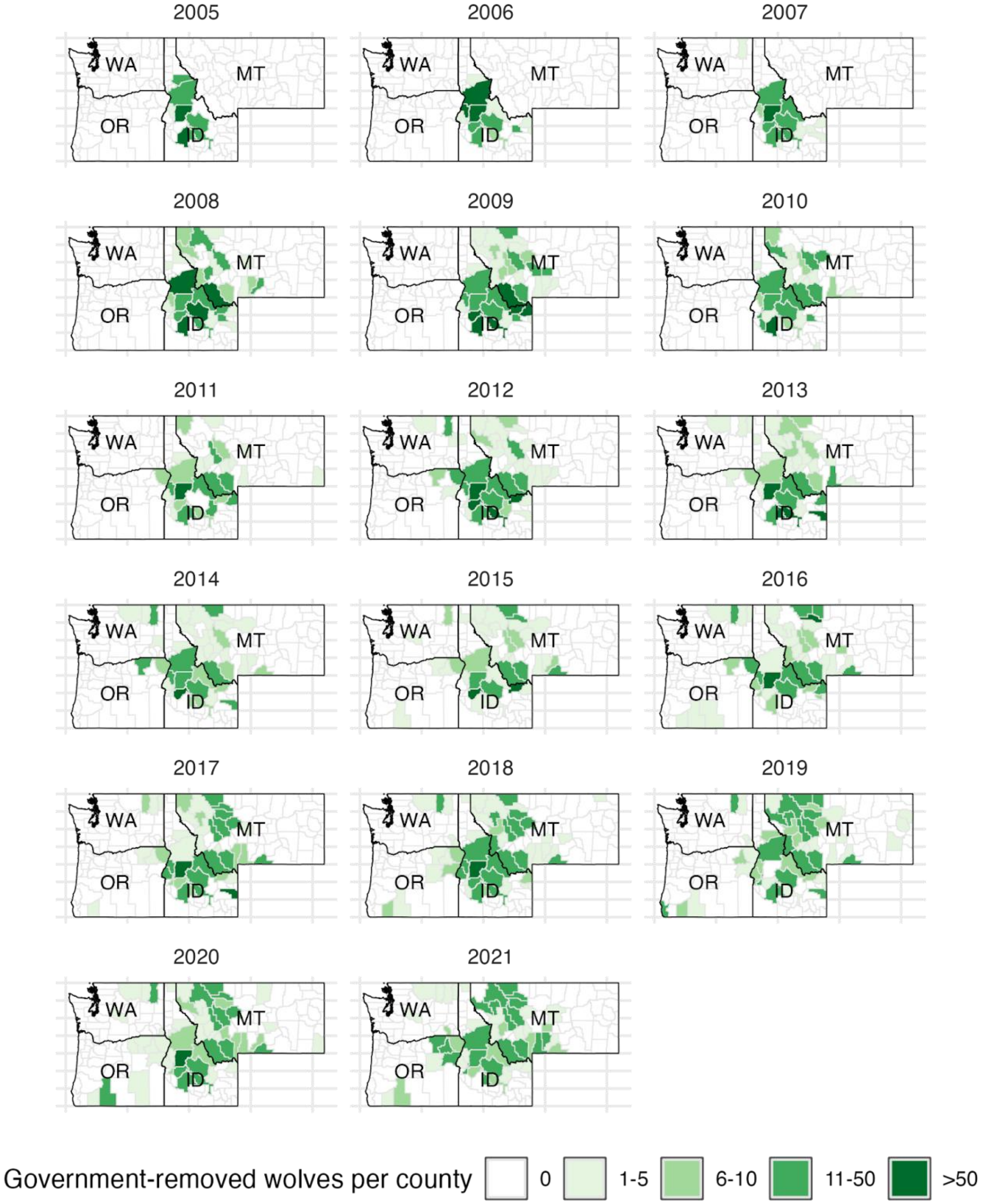

Fig. S4.

Distribution of wolves lethally removed by government agencies or government-removed wolves across counties and over time. Darker colors represent higher numbers of removed wolves.

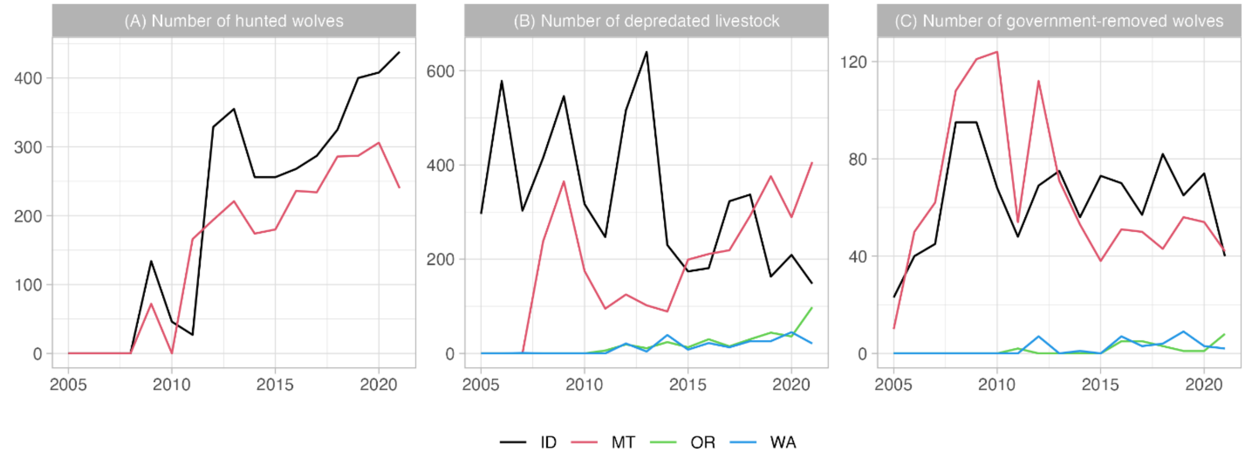

Fig. S5.

State-level over-time trends of hunted wolves (panel A), livestock depredation by wolves (panel B) and government wolf removals (panel C).

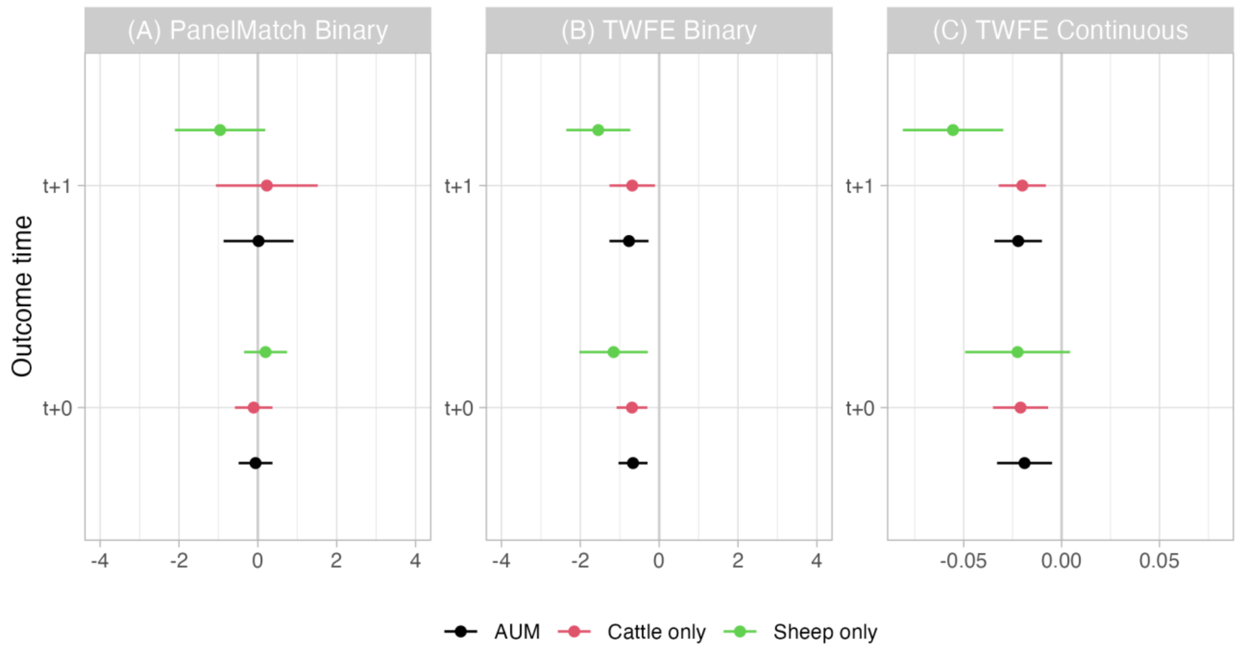

Fig. S6.

Effects of wolf hunting on livestock depredation with alternative livestock measures of Sheep only in blue, cattle only in green and AUM (animal unit month) in orange for difference-in differences panel modeling including PanelMatch Binary (panel A), two-way-fixed effect binary (panel B), and two-way fixed effect continuous (panel C) for the same year t+0 and the following year t+1. AUM is a common way of standardizing forage needs of different livestock types.

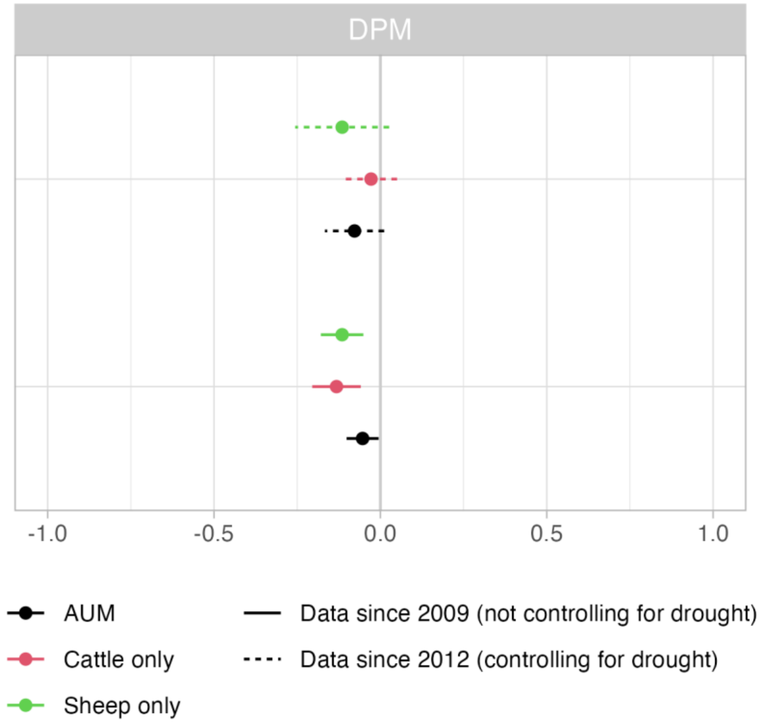

Fig. S7.

Effects of wolf hunting on livestock depredation with alternative livestock measures of sheep only in blue, cattle only in green, and AUM (animal unit month) in orange for DPM (dynamic panel modeling). AUM is a common way of standardizing forage needs of different livestock types. Solid lines indicate data since 2009 not controlling for drought while dashed lines indicate data since 2012 while controlling for drought.

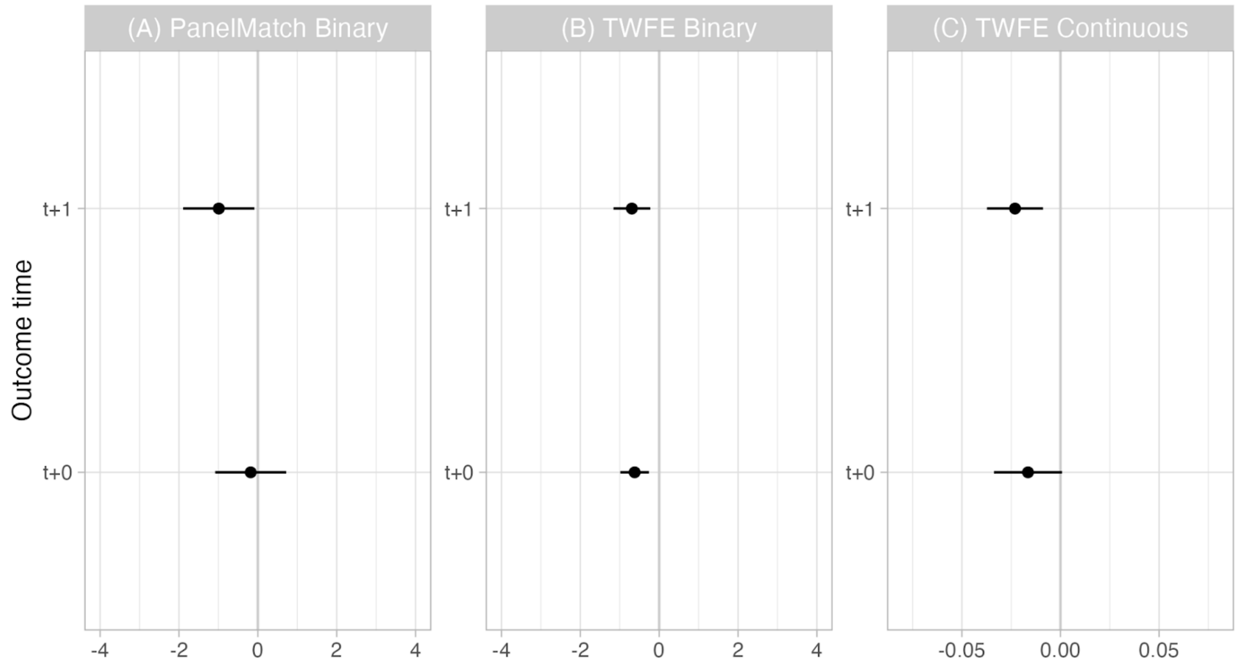

Fig. S8. Effects of wolf hunting on livestock depredation for Montana and Idaho only for difference-in differences panel modeling including PanelMatch binary (panel A), two-way fixed effect binary (panel B), and two-way fixed effect continuous (panel C) for the same year t+0 and the following year t+1.

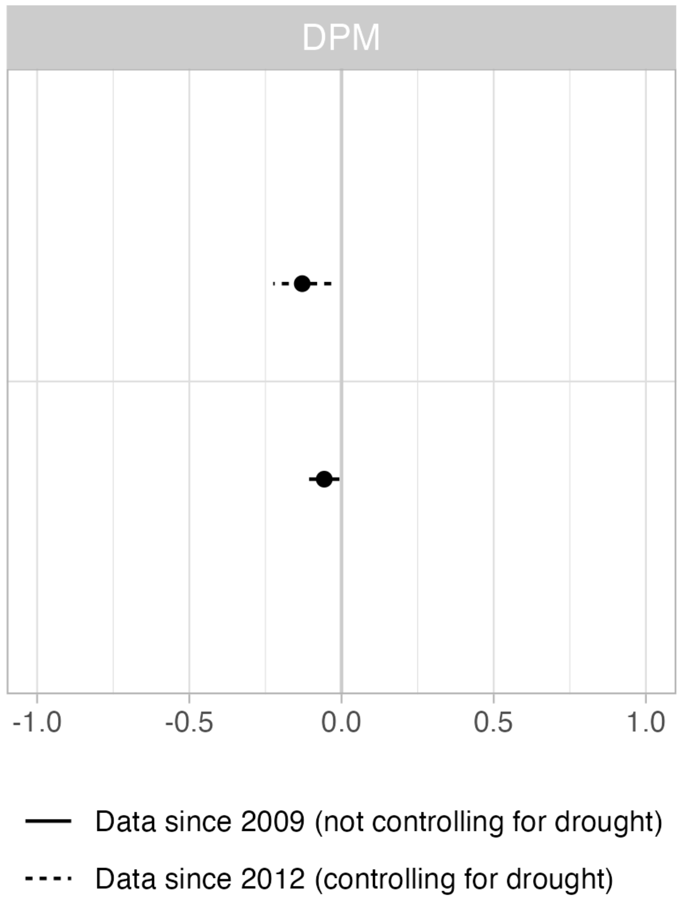

Fig. S9.

Effects of wolf hunting on livestock depredation for Montana and Idaho only using DPM (dynamic panel modeling). Solid lines indicate data since 2009 not controlling for drought and dashed lines indicate data since 2012 controlling for drought.

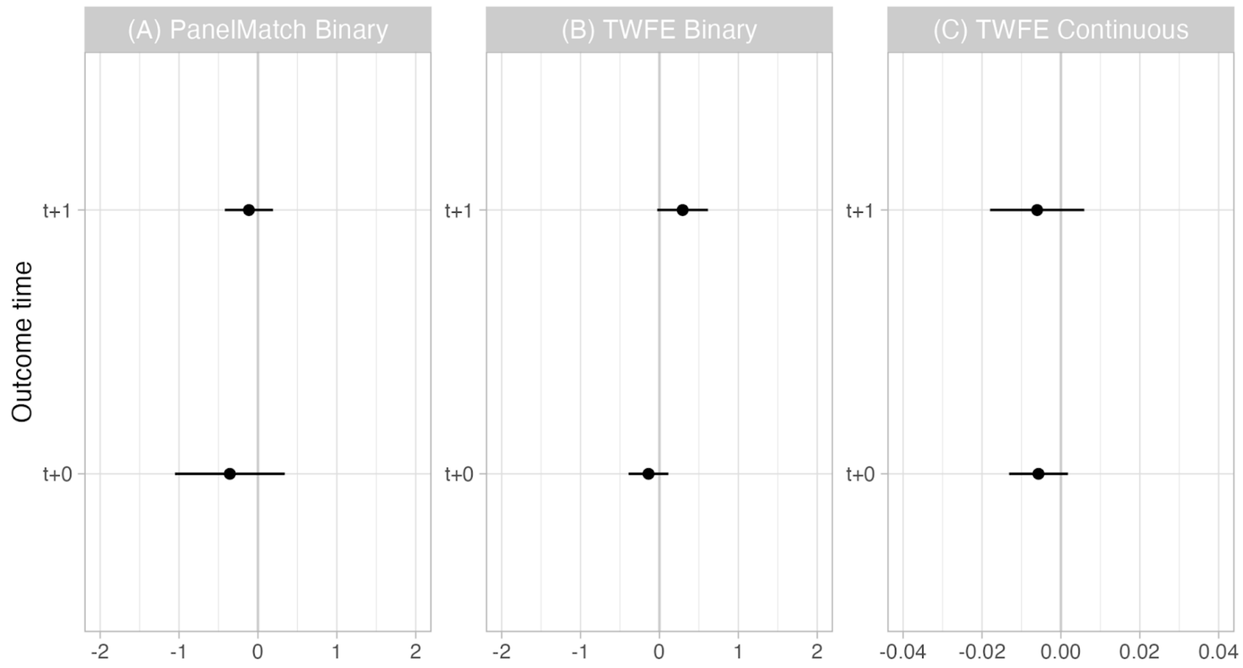

Fig. S10.
Effects of wolf hunting on government removal, for Montana and Idaho only for difference-in differences panel modeling including PanelMatch binary (panel A), two-way fixed effect binary (panel B), and two-way fixed effect continuous (panel C) for the same year t+0 and the following year t+1.

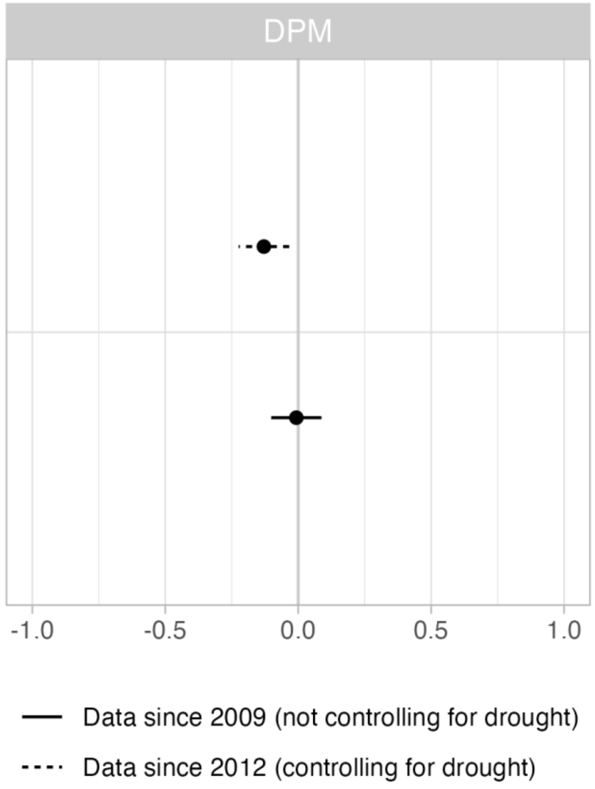

Fig. S11.

Effects of wolf hunting on government removal, for Montana and Idaho only for DPM (dynamic panel modeling). Solid lines indicate data since 2009 not controlling for drought and dashed lines indicate data since 2012 controlling for drought.

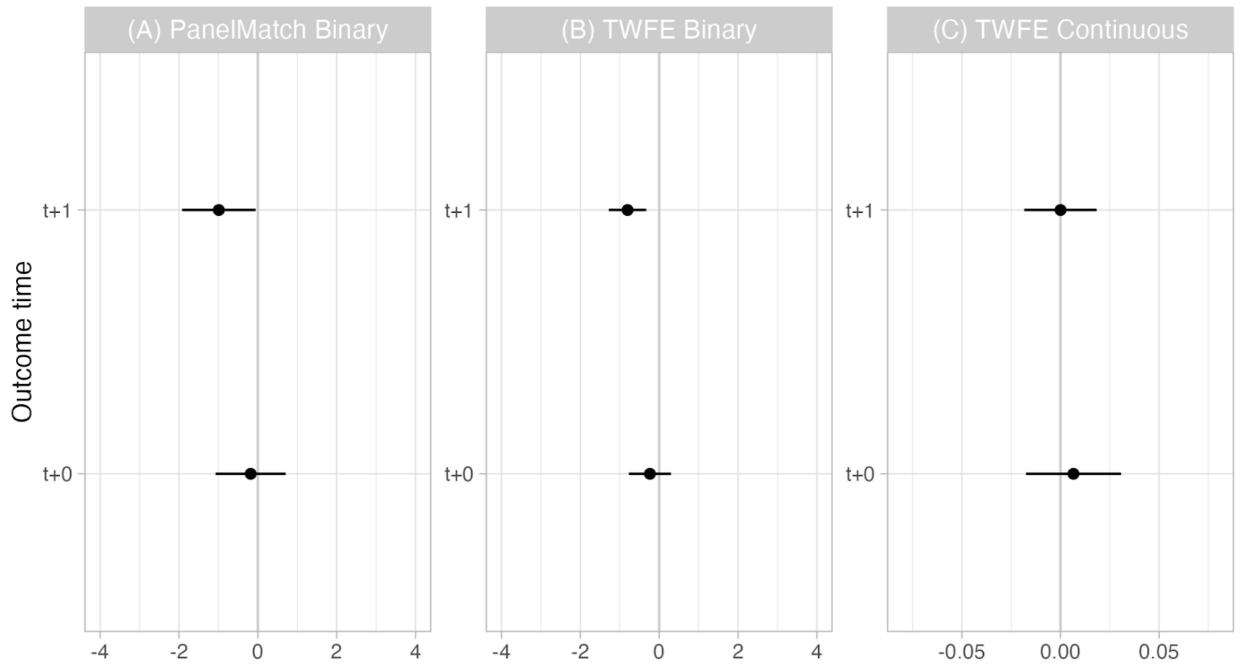

Fig. S12.

Effects of wolf hunting on livestock depredation for 2012 and later for difference-in differences panel modeling including PanelMatch binary (panel A), two-way fixed effect binary (panel B), and two-way fixed effect continuous (panel C) for the same year t+0 and the following year t+1.

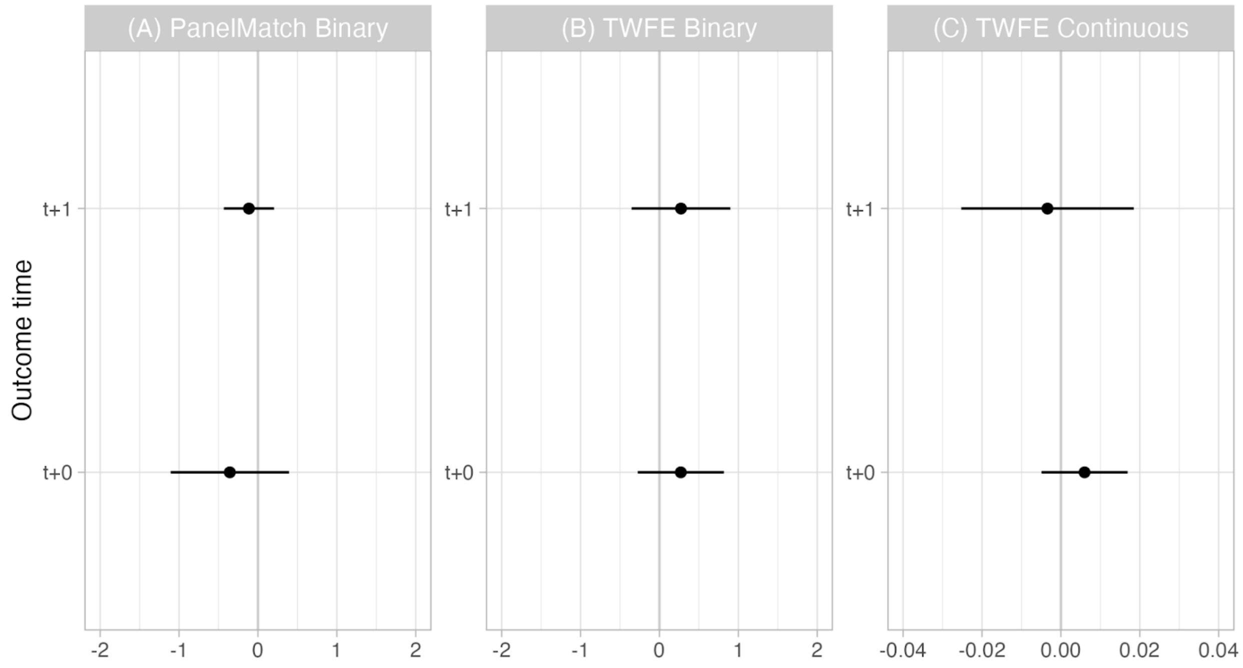

Fig. S13.

Effects of wolf hunting on government removal for 2012 and later for difference-in differences panel modeling including PanelMatch binary (panel A), two-way fixed effect binary (panel B), and two-way fixed effect continuous (panel C) for the same year t+0 and the following year t+1.

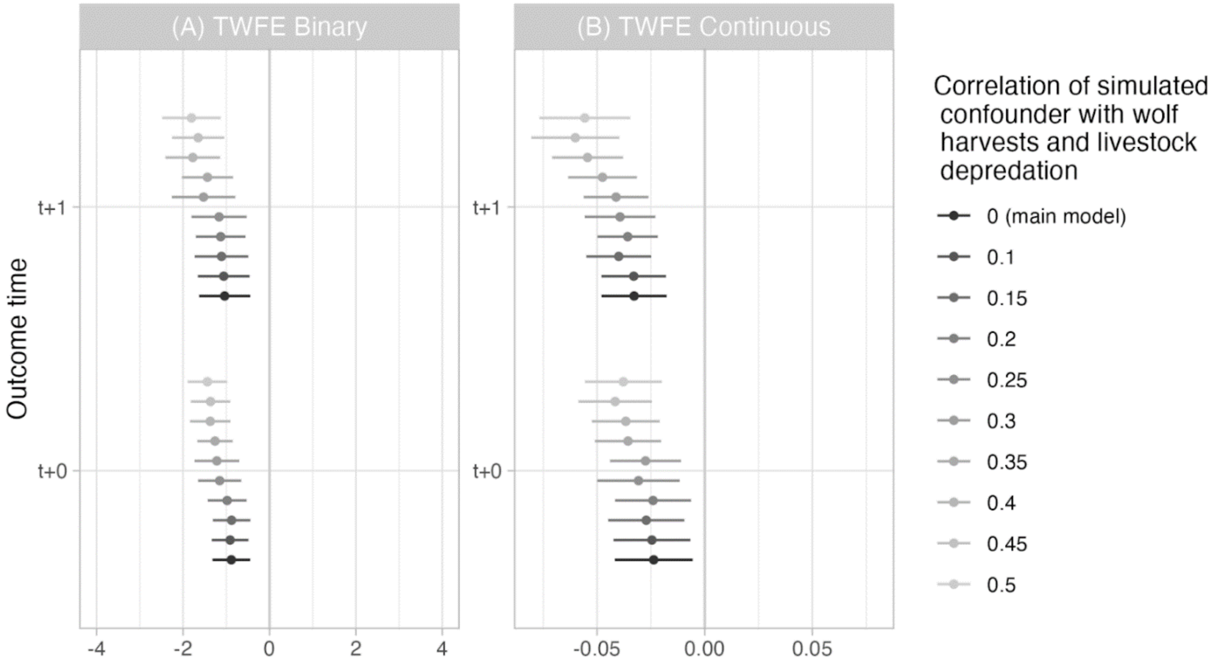

Fig. S14.

Effects of wolf hunting on livestock depredation when simulating a confounder correlated to both predictor and outcome for binary (Panel A) and continuous (Panel B) two-way fixed effect models.
